# Late-Onset Li-Fraumeni Syndrome-Like Phenotype Presenting With Synchronous Lung Adenocarcinoma and Ovarian High-Grade Serous Carcinoma: A Case Report

**DOI:** 10.7759/cureus.102130

**Published:** 2026-01-23

**Authors:** Naga Lakshmi Gannavaram, Yousef Shweihat, Nadim Bou Zgheib, Krista L Denning, John Diks

**Affiliations:** 1 Obstetrics and Gynecology, Marshall University Joan C. Edwards School of Medicine, Huntington, USA; 2 Internal Medicine, Marshall University Joan C. Edwards School of Medicine, Huntington, USA; 3 Pathology, Marshall University Joan C. Edwards School of Medicine, Huntington, USA

**Keywords:** cancer predisposition syndrome, hereditary cancer, li-fraumeni-like syndrome, li-fraumeni syndrome, mosaic tp53 mutation, p53 tetramerization domain, tp53 f341y, tp53 mutation

## Abstract

Li-Fraumeni syndrome is classically defined by germline *TP53* mutations and early-onset malignancies, yet the clinical spectrum has expanded to include atypical and late-onset presentations that challenge standard diagnostic criteria. We report the case of a 53-year-old female diagnosed with synchronous high-grade serous ovarian carcinoma and lung adenocarcinoma. Somatic analysis identified *TP53* driver mutations in both tumors, including a specific F341Y variant, despite negative comprehensive germline testing. This discordance suggests a mosaic somatic mutation or a Li-Fraumeni-like phenotype distinct from classic inheritance patterns. This case illustrates the complexities of diagnosing hereditary cancer syndromes in patients with multiple primary malignancies and highlights the importance of integrating tumor genomic profiling with germline analysis to ensure accurate risk stratification and clinical management.

## Introduction

Li-Fraumeni syndrome (LFS) is a rare, autosomal dominant hereditary cancer predisposition syndrome characterized by a high lifetime risk of a wide spectrum of cancers, often occurring at an early age [1]. The classic LFS-defining cancers include soft tissue sarcomas, osteosarcomas, premenopausal breast cancer, brain tumors, and adrenocortical carcinomas [2]. The genetic basis of LFS is primarily attributed to germline mutations in the TP53 tumor suppressor gene, which plays a critical role in cell cycle regulation, DNA repair, and apoptosis [3]. Additionally, impairments in the cell protective mechanisms are unchecked, thus promoting tumorigenesis [4]. LFS is associated with an increased risk of ovarian cancer, though this risk is lower than that of the hallmark malignancies [5]. Importantly, high-grade serous carcinoma (HGSC) is universally driven by TP53 mutations and is the most common form of epithelial ovarian cancer [6].

While the classic LFS definition provides a framework for identifying affected families, it is increasingly recognized that the clinical spectrum of TP53-related cancers is broader than initially described. This has led to the concept of the Li-Fraumeni spectrum, which encompasses a range of phenotypes, from classic LFS to attenuated or Li-Fraumeni-like (LFL) syndromes, where individuals or families may not meet the strict diagnostic criteria but still carry pathogenic TP53 variants [3]. These individuals may present with later-onset cancers or a diﬀerent tumor profile than those with classic LFS. Classic LFS diagnosis is outlined by the Chompret criteria, which emphasizes an early-onset sarcoma, multiple tumors in the proband or in close relatives, alongside the presence of a pathogenic germline TP53 mutation [7]. By contrast, LFL patients share similar case presentations but do not fully meet the clinical and pathogenic guidelines for LFS [8]. Such patients may present with later-onset malignancies or non-classical tumor types. For example, lung adenocarcinoma is not a hallmark LFS malignancy, but it is associated with somatic TP53 mutations, illustrating the heterogeneity of TP53-driven pathogenesis in cancer [9].

Primary malignancies can occur metachronously, when tumors occur six or more months apart, and synchronously, where malignancies can occur within six months of each other [10]. Patients with LFS are at risk for multiple malignancies, which typically occur metachronously. In comparison, the synchronous presentation of two TP53-driven malignancies is relatively rare, as most carriers develop malignancies sequentially [11]. The synchronous occurrence of distinct TP53-driven malignancies underscores the heterogeneity of TP53-related oncogenesis. Accurately differentiating between the variations of primary tumor pathogenesis from metastatic disease is important, as it can impact staging, prognosis, and treatment options [12].

Case reports further illustrate the variability in the Li-Fraumeni spectrum. One woman presented with a familial history of malignancies and a TP53 mutation, with breast cancer at age 48 [1]. Another case report described a patient with early-onset malignancies but no strong family history, who was later found to carry a TP53 mutation [13]. Together, both of these cases underscore the heterogeneity of TP53 mutations and how atypical presentations can still fall within the Li-Fraumeni spectrum.

We describe a 53-year-old female with synchronous TP53-driven ovarian and lung malignancies who responded favorably to chemotherapy. Despite her older age, this atypical presentation underscores the expanding Li-Fraumeni spectrum and the importance of genetic testing in synchronous cancers, even without classic early-onset features.

## Case presentation

Patient profile and history of present illness 

A 53-year-old Caucasian female initially presented with a large, complex left ovarian cyst accompanied by an elevated CA-125 level. At the time of admission, she denied constitutional symptoms, such as fevers, chills, or weight loss, and reported no pain. Her medical history was otherwise significant for a prior tubal ligation and a left breast lumpectomy. She is a former smoker who quit more than 30 days prior to initial presentation. Despite a detailed assessment of the family history, no clinically significant history of cancer was identified.

Clinical course and management (month 0)

**Figure 1:**
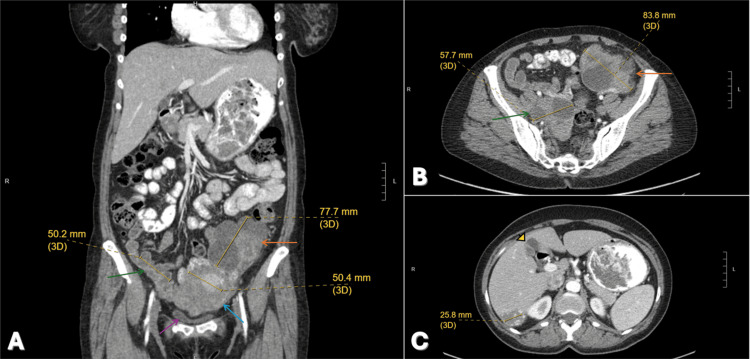
Coronal CT view of the patient A) Coronal CT view showing the uterus and ovaries are abnormal, with the uterus (blue arrow) containing multiple nodules and masses measuring up to 5.7 cm in diameter, with mass effects on the urinary bladder (purple arrow), bilateral adnexal masses (orange and green arrows), and free fluid (ascites) present in the pelvis. B) Axial CT view showing multiple simple cysts within the bilateral ovaries. The ovaries are enlarged, with the left measuring 9.8 x 6.6 cm in diameter (orange arrow) and the right 7.3 x 4.7 cm in diameter (green arrow). C) Axial CT view showing liver with two rounded hypodense nodules within the superior aspect of the right hepatic lobe, most likely cysts (arrowhead). There is a 3.5 cm right hepatic lobe hemangioma.

The patient underwent an exam under anesthesia, diagnostic laparoscopy, and a robotic-assisted total laparoscopic hysterectomy with bilateral salpingo-oophorectomy (RATLH/BSO). Intraoperative findings revealed a large left ovarian mass measuring 10-15 cm with extensive tumor nodularity extending to the pelvic peritoneum, posterior cul-de-sac, and bladder peritoneum. An enlarged right ovarian complex cyst (4-5 cm) and moderate volume of ascites were also noted (Figures 1A-1B).

Surgical pathology and molecular profile 

**Figure 2:**
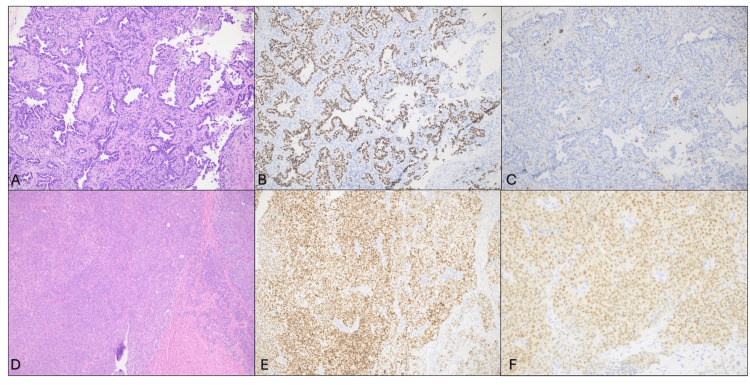
Histopathologic examination of the patient A) H&E x100 showing lung adenocarcinoma with lepidic pattern. B) TTF-1 IHC x100 is positive in lung adenocarcinoma cells. C) PAX-8 IHC x100 is negative in lung adenocarcinoma cells. D) H&E x100 showing ovarian high-grade serous carcinoma. E) TP53 IHC x100 is positive (diffuse mutant pattern). F) PAX-8 IHC x200 is positive ovarian serous carcinoma cells.​

Histopathologic examination confirmed high-grade serous carcinoma involving bilateral ovaries with surface involvement. The disease was staged as pT3c pNX (International Federation of Gynecology and Obstetrics (FIGO) IIIC) due to macroscopic peritoneal metastasis beyond the pelvis greater than 2 cm. Immunohistochemistry showed the malignant cells were positive for WT1, PAX8, and CK7, with overexpression of p53 while being negative for CK20 and CDX2 (Figure 2).

Months 1-6: Adjuvant therapy and surveillance 

Following surgery, the patient completed six cycles of adjuvant chemotherapy with carboplatin and paclitaxel. During this period, she experienced fatigue and constipation but denied neuropathy or other severe adverse effects. Comprehensive genetic testing using a 77-gene panel (including BRCA1, BRCA2, and TP53) was performed, but no deleterious germline mutations were identified at that time. Initial staging chest imaging noted scattered 2-3 mm subpleural pulmonary nodules, which were deemed indeterminate and monitored.

Years 3-5: Disease progression and second malignancy 

**Figure 3:**
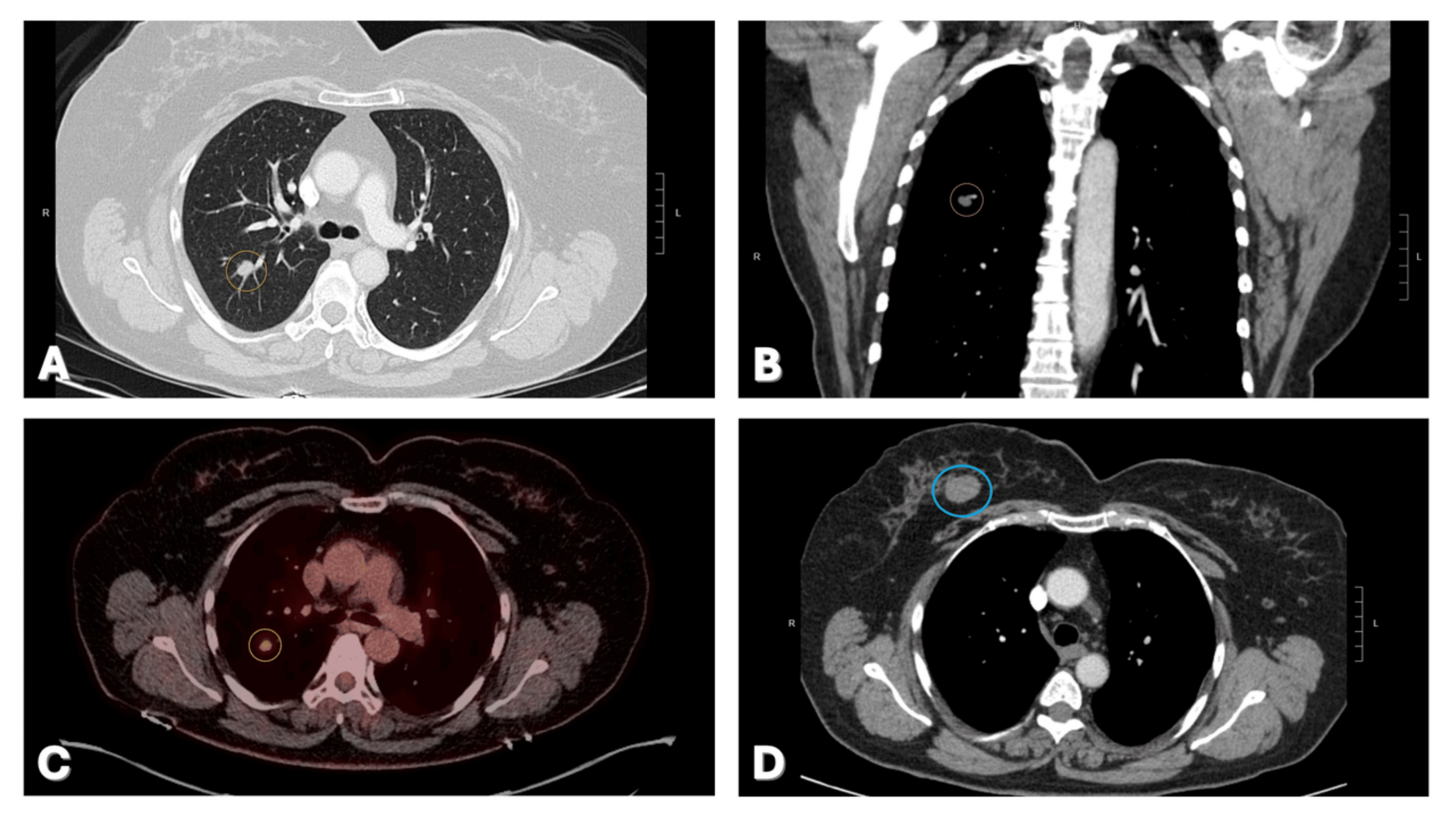
Patient's routine surveillance imaging A) Axial CT view showing the oval, 1.1 cm x 0.9 cm, right upper lobe pulmonary nodule (yellow circle). B) Coronal CT view showing the right upper lobe lung nodule (yellow circle). C) PET/ CT scan showing the hypermetabolic right upper lobe nodule (SUV of 4.6) (yellow circle). D) Axial CT view showing the 2.5 cm nodule upper central right breast (blue circle).

Routine surveillance imaging initially showed stability; however, by year 3, a specific nodule in the right upper lobe began to slowly enlarge, growing from 2-3 mm to approximately 9 mm and reached 1.1 cm by year 5 (Figures 3A-3B). A PET/CT scan revealed this nodule to be hypermetabolic (Figure 3C), while no FDG-avid thoracic lymphadenopathy was present. A subsequent biopsy confirmed a diagnosis of lung adenocarcinoma with a lepidic growth pattern (Figure 2). Comparative molecular analysis revealed that both the new lung adenocarcinoma and the prior ovarian high-grade serous carcinoma harbored TP53 mutations. This concordance of TP53 mutations in synchronous/metachronous dual primaries raised suspicion for a Li-Fraumeni or LFL syndrome phenotype, despite the previous negative germline screening, prompting a recommendation for re-evaluation of genetic status. 

Additional findings

Radiologic evaluation consistently identified a hemangioma located in the posterior right hepatic lobe. During the initial staging period (around month 0), this lesion measured 3.9 x 2.4 cm. Follow-up imaging several years later described the lesion as an enhancing mass measuring approximately 2.7 cm in diameter (Figure 1C). Throughout the surveillance period, the hemangioma remained stable with no new or enlarging lesions noted. In addition to the hemangioma, the patient was noted to have multiple hepatic cysts and profound hepatic steatosis (fatty liver).

The patient had a persistent, rounded mass in the right breast, frequently noted in imaging reports. During the initial chemotherapy course (month 3), the mass measured 2.8 x 2.2 cm and was characterized as compatible with fibroadenomas and cysts. In later surveillance (years 3-5), the mass remained stable, measuring between 2.3 cm and 2.5 cm (Figure 3D). A PET scan performed during the workup for the lung nodule showed only low-level radiotracer uptake in this 2.3 cm mass. The "low-level uptake" on PET/CT, in conjunction with long-term radiographic stability (years 3-5), strongly supports a benign etiology such as a fibroadenoma, rather than a malignancy, which would typically demonstrate hypermetabolism (high SUV) and interval growth.

Laboratory results

Table 1 summarizes the patient's laboratory values obtained during the course of adjuvant chemotherapy, indicating preserved organ function and hematologic stability. 

**Table 1:** The patient's laboratory values ALT: alanine transaminase; AST: aspartate aminotransferase; BUN: blood urea nitrogen

Laboratory Test	Patient Result	Normal Reference Range
Hematology		
White Blood Cell (WBC)	4.8	4.5-11.0 k/cmm
Hemoglobin (Hgb)	13.8	12.0-15.5 gm/dL
Hematocrit (Hct)	40.6	37.0-47.0%
Platelets	179	150-400 k/cmm
Neutrophils (Absolute)	2.7	1.8-7.7 k/cmm
Lymphocytes (Absolute)	1.5	1.0-4.8 k/cmm
Chemistry		
Potassium	4	3.5-5.0 mEq/L
Chloride	106	98-107 mEq/L
Glucose	8	70-100 mg/dL
BUN	14	6-20 mg/dL
Creatinine	0.74	0.6-1.1 mg/dL
Calcium	9.1	8.5-10.5 mg/dL
Liver Function		
Alkaline Phosphatase	67	44-147 IU/L
ALT (SGPT)	40	7-56 IU/L
AST (SGOT)	19	10-40 IU/L

## Discussion

This case report describes a 53-year-old female with stage IIIC ovarian cancer who has responded favorably to chemotherapy. While ovarian cancer is a common malignancy, the patient's age at diagnosis, coupled with the detailed clinical and genetic information (BRCA-negative, PDLl-positive, high genomic loss of heterozygosity (LOH)), prompts a deeper consideration of potential underlying genetic predispositions, particularly within the evolving understanding of the Li-Fraumeni spectrum.

LFS is a well-established hereditary cancer syndrome primarily caused by germline mutations in the TP53 tumor suppressor gene. The TP53 gene encodes the p53 protein, a critical regulator of cell growth and division, often referred to as the "guardian of the genome" [3]. Pathogenic TP53 variants significantly increase the lifetime risk of developing various cancers, often at an early age. However, the phenotypic expression of TP53 mutations is increasingly recognized as heterogeneous, leading to the concept of the Li-Fraumeni spectrum [4]. This spectrum includes classic LFS, characterized by speciﬁc early-onset cancers (e.g., sarcomas, premenopausal breast cancer, brain tumors, adrenocortical carcinomas), and LFL syndromes, which encompass a broader range of cancer types and later ages of onset [1,4].

The provided literature sheds light on this expanded spectrum. Kratz et al. [2] analyzed an international germline TP53 variant dataset, proposing a classiﬁcation for the Li- Fraumeni spectrum that includes (l) phenotypic LFS (meeting clinical criteria without a TP53 variant), (2) LFS (meeting testing criteria with a TP53 variant), (3) attenuated LFS (having a TP53 variant but not meeting LFS testing criteria, often with later-onset cancers), and (4) incidental LFS (having a TP53 variant without a cancer history). Their findings indicate signiﬁcant difference in tumor spectra between those who meet LFS genetic testing criteria and those who do not. Notably, carriers who did not meet LFS criteria had a higher incidence of breast cancer and other cancers, with 45% occurring after age 45 years [2]. This observation is particularly relevant to our patient, whose ovarian cancer diagnosis at age 53 falls within this later-onset category, suggesting a possible attenuated LFS phenotype.

Cho et al. [1] present a case of late-onset LFL syndrome with unilateral breast cancer, featuring a germline TP53 missense mutation (c.566C>T, Al89V). This mutation had been previously reported in a case of multiple primary colon tumors with late onset. The authors highlight that neither of these cases fulﬁlled previously deﬁned criteria for LFS due to the relatively late onset of malignancies. They suggest that the Al89V mutation may have a relatively weak dominant-negative effect compared to other TP53 mutations, contributing to the later onset of cancer. This further supports the idea that TP53 mutations can manifest with varying penetrance and age of onset, extending beyond the classic LFS presentation.

Zhuang et al. [3] describe a case of an LFS-like phenotype with a de novo mutation in CHEK2, another gene associated with cancer predisposition. While their case focuses on CHEK2 rather than TP53, it underscores the complexity of hereditary cancer syndromes and the potential for other genetic factors to mimic or contribute to LFS-like presentations. The presence of high genomic LOH in our patient, even with a negative BRCA result, suggests that other genetic alterations may be at play, warranting further investigation into a broader panel of cancer predisposition genes.

Another important aspect of this case is the potential for diagnostic and staging errors when multiple lesions are identified. Without proper biopsy confirmation, multiple independent primary malignancies can mimic widespread metastasis. In one study, among lesions presumed to be metastatic, approximately 14% were discovered to be either secondary primary tumors or benign upon biopsy, further underscoring the importance of histological classification [13]. Misclassifying synchronous primaries as metastatic disease can lead to improper palliative management and delay proper therapy options. For example, in a reported case of concurrent lung and liver lesions, initial biopsy results were inconclusive, but with further histological testing, imaging, and genetic analysis, the subsequent liver biopsy was confirmed to be a synchronous primary carcinoma, hence modifying the existing treatment plan [14,15]. This emphasizes the necessity of comprehensive diagnostic evaluation, including biopsy and molecular analysis, to ensure effective treatment plans.

Our patient's favorable response to chemotherapy, despite advanced-stage disease, is also noteworthy. While the literature on TP53 mutations and chemotherapy response is complex and sometimes contradictory, some studies suggest that certain TP53 alterations can inﬂuence treatment outcomes. The high genomic LOH observed in our patient could be a factor inﬂuencing her response, as it may indicate a higher degree of genomic instability that could make the tumor more susceptible to DNA-damaging agents such as carboplatin and paclitaxel. Further research is needed to fully elucidate the interplay between specific TP53 variants, other genetic alterations, and treatment response in ovarian cancer. In conclusion, this case illustrates the expanding clinical spectrum of TP53-related cancers and the importance of considering attenuated or LFL syndromes in patients with later-onset malignancies. The favorable response to chemotherapy in this patient, combined with her genetic profile, highlights the need for comprehensive genetic counseling and testing in individuals with a strong personal or family history of cancer, even if they do not meet the classic LFS criteria. A deeper understanding of the diverse manifestations of TP53 mutations and other cancer predisposition genes will be crucial for personalized risk assessment, early detection, and tailored therapeutic strategies.

## Conclusions

In conclusion, this case illustrates a rare synchronous presentation of two TP53-driven mutations of high-grade, serious ovarian carcinoma and lung adenocarcinoma. Awareness of such presentations can expand our understanding of the LFS spectrum, emphasizing that TP53-related malignancies can have synchronous presentation and an onset of malignancy at a later age than the traditional early-onset of Li-Fraumeni Syndrome. Comprehensive genetic and molecular testing can be useful in the diagnosis of patients with both late-onset and atypical malignancy presentations. Without proper diagnostics, these presentations can be misinterpreted as advanced metastatic cancer, which can result in improper treatment and patient management. Ultimately, this case illustrates the importance of maintaining suspicion for TP53-related malignancies, such as in older patients without a family history of malignancies. We acknowledge the inherent limitations of a single case study and the technical challenges in definitively confirming somatic mosaicism. Future collection of similar cases is necessary to fully characterize the clinical implications of these atypical TP53 presentations, refining the Li-Fraumeni spectrum classification system and advancing oncology approaches.
